# Correction: Dietary patterns of adults in Italy: Results from the third Italian National Food Consumption Survey, INRAN-SCAI

**DOI:** 10.1371/journal.pone.0341477

**Published:** 2026-01-20

**Authors:** The PLOS One Staff

The Funding statement for this article is incorrect. The correct Funding statement is as follows: This work is part of the PhD project of Nicolò Scarsi at Università Cattolica del Sacro Cuore, funded under the PON ‘Research and Innovation’ 2014–2020 – Action IV.5 ‘Doctorates on Green Issues’. The project is titled ‘Development of a Tool to Assess Adherence to the Planetary Health Diet Proposed by the EAT-Lancet Commission’. The funder had no role in the establishment of the present work. Università Cattolica del Sacro Cuore contributed to the funding of this research project and its publication (Linea D3.1 2023).

There are errors in the Author Contributions. The correct contributions are:

**Conceptualization:** Nicolò Scarsi, Roberta Pastorino, Angelo Maria Pezzullo, Stefania Boccia

**Data curation:** Nicolò Scarsi, Cosimo Savoia, Gian Marco Raspolini, Angelo Maria Pezzullo

**Formal analysis:** Nicolò Scarsi, Angelo Maria Pezzullo

**Funding acquisition:** Stefania Boccia

**Investigation:** Nicolò Scarsi, Roberta Pastorino, Cosimo Savoia, Gian Marco Raspolini, Angelo Maria Pezzullo, Stefania Boccia

**Methodology:** Nicolò Scarsi, Roberta Pastorino, Angelo Maria Pezzullo, Stefania Boccia

**Project administration:** Nicolò Scarsi, Roberta Pastorino, Angelo Maria Pezzullo, Stefania Boccia

**Resources:** Stefania Boccia

**Software:** Nicolò Scarsi

**Supervision:** Roberta Pastorino, Angelo Maria Pezzullo, Stefania Boccia

**Validation:** Nicolò Scarsi, Roberta Pastorino, Angelo Maria Pezzullo, Stefania Boccia

**Visualization:** Nicolò Scarsi, Roberta Pastorino, Angelo Maria Pezzullo

**Writing – original draft:** Nicolò Scarsi, Roberta Pastorino, Cosimo Savoia, Gian Marco Raspolini, Angelo Maria Pezzullo

**Writing – review & editing:** Nicolò Scarsi, Roberta Pastorino, Angelo Maria Pezzullo, Stefania Boccia

The publisher apologizes for the errors.
