# Supplementary material for: Dietary patterns of adults in Italy: Results from the third Italian National Food Consumption Survey, INRAN-SCAI
Source: PLoS One. 2025 Jun 13;20(6):e0312977. doi: 10.1371/journal.pone.0312977 (PMC12165367; doi:10.1371/journal.pone.0312977)
Supplement: S1 Table — (DOCX) [file pone.0312977.s001.docx]

**S1 Table.** Estimated factor loadings from principal component analysis.

| **Food groups** | **PC1** | **PC2** | **PC3** | **PC4** | **PC5** |
| --- | --- | --- | --- | --- | --- |
|  |  |  |  |  |  |
| Grain products | * | * | * | 0.6115 | * |
| Vegetables | 0.3237 | * | 0.2573 | 0.2112 | -0.3054 |
| Starchy roots | 0.2444 | * | * | * | * |
| Legumes | * | * | * | * | * |
| Fruits | * | * | 0.4367 | * | * |
| Pork, not preserved, excl. offal | * | * | -0.2528 | * | * |
| Processed meat | * | 0.3795 | * | * | * |
| Poultry and game, not preserved, excl. offal | * | * | * | * | * |
| Beef and veal, not preserved, excl. offal | 0.4074 | * | * | * | * |
| Offals, blood, and their product | * | 0.4120 | * | * | * |
| Other meats, not preserved, excl. offal | * | 0.4382 | * | * | * |
| Fish | * | * | * | * | * |
| Dairy products | * | * | * | * | * |
| Eggs products | 0.3464 | * | * | * | * |
| Sugar and confectionary | * | * | * | * | 0.6946 |
| Fat and oils | 0.5401 | * | * | * | * |
| Juices | * | 0.2681 | * | * | * |
| Non-alcoholic beverages | * | * | * | * | 0.5599 |
| Alcoholic beverages | 0.2372 | * | -0.4654 | * | * |
| Water | * | * | 0.5941 | * | * |
| Herbs, spices and condiments | * | * | * | 0.6290 | * |
| Special nutritional | * | -0.4115 | * | * | * |
| Composite food | * | 0.2611 | * | * | * |
| Snacks etc. | * | 0.3569 | * | * | * |

*** Food groups with a factor loading between −0.20 and 0.20 were not showed

Factor loadings pictures the correlations between the original variables (food groups) and the principal components (dietary patterns). They tell us how much each variable matters to a particular component. The sign of the loadings (positive or negative) suggests the direction of such relation. A positive loading means the variable positively contributes to that principal component, while the negative one is explanatory of an inverse relationship. Higher absolute values (closer to 1) suggest a stronger relationship, those variables with higher loadings (either positive or negative) have a bigger impact on shaping that component.
