# Supplementary material for: Dietary patterns of adults in Italy: Results from the third Italian National Food Consumption Survey, INRAN-SCAI
Source: PLoS One. 2025 Jun 13;20(6):e0312977. doi: 10.1371/journal.pone.0312977 (PMC12165367; doi:10.1371/journal.pone.0312977)
Supplement: S2 Table — (DOCX) [file pone.0312977.s002.docx]

**S2 Table**. Individuals showing high adherence (Z-Score ≥1) to multiple dietary patterns.

| **COMBINATION** | **PC1** | **PC2** | **PC3** | **PC4** | **PC5** | **INDIVIDUALS** | **%** |
| --- | --- | --- | --- | --- | --- | --- | --- |
| 1 | **X** | **X** | **X** | **X** | **X** | 10 | 0.83 |
| 2 | **X** | **X** | **X** | **X** |  | 19 | 1.58 |
| 3 | **X** | **X** | **X** |  | **X** | 22 | 1.83 |
| 4 | **X** | **X** | **X** |  |  | 49 | 4.08 |
| 5 | **X** | **X** |  | **X** | **X** | 11 | 0.92 |
| 6 | **X** | **X** |  | **X** |  | 22 | 1.83 |
| 7 | **X** | **X** |  |  | **X** | 16 | 1.33 |
| 8 | **X** | **X** |  |  |  | 33 | 2.75 |
| 9 | **X** |  | **X** | **X** | **X** | 2 | 0.17 |
| 10 | **X** |  | **X** | **X** |  | 10 | 0.83 |
| 11 | **X** |  | **X** |  | **X** | 5 | 0.42 |
| 12 | **X** |  | **X** |  |  | 17 | 1.41 |
| 13 | **X** |  |  | **X** | **X** | 9 | 0.75 |
| 14 | **X** |  |  | **X** |  | 58 | 4.83 |
| 15 | **X** |  |  |  | **X** | 35 | 2.91 |
| 16 | **X** |  |  |  |  | 92 | 7.65 |
| 17 |  | **X** | **X** | **X** | **X** | 1 | 0.08 |
| 18 |  | **X** | **X** | **X** |  | 10 | 0.83 |
| 19 |  | **X** | **X** |  | **X** | 16 | 1.33 |
| 20 |  | **X** | **X** |  |  | 63 | 5.24 |
| 21 |  | **X** |  | **X** |  | 12 | 1.00 |
| 22 |  | **X** |  |  | **X** | 18 | 1.50 |
| 23 |  | **X** |  |  |  | 65 | 5.41 |
| 24 |  |  | **X** | **X** |  | 16 | 1.33 |
| 25 |  |  | **X** |  | **X** | 10 | 0.83 |
| 26 |  |  | **X** |  |  | 119 | 9.90 |
| 27 |  |  |  | **X** | **X** | 21 | 1.75 |
| 28 |  |  |  | **X** |  | 221 | 18.39 |
| 29 |  |  |  |  | **X** | 216 | 17.97 |
| 30 |  | **X** |  | **X** | **X** | 4 | 0.33 |
| Total |  |  |  |  |  | **1.202** |  |
